# Determination of sRNA Expressions by RNA-seq in *Yersinia pestis* Grown *In Vitro* and during Infection

**DOI:** 10.1371/journal.pone.0074495

**Published:** 2013-09-11

**Authors:** Yanfeng Yan, Shanchun Su, Xiangrong Meng, Xiaolan Ji, Yi Qu, Zizhong Liu, Xiaoyi Wang, Yujun Cui, Zhongliang Deng, Dongsheng Zhou, Wencan Jiang, Ruifu Yang, Yanping Han

**Affiliations:** 1 State Key Laboratory of Pathogen and Biosecurity, Beijing Institute of Microbiology and Epidemiology, Beijing, China; 2 Microbiology Laboratory, Sichuan Agricultural University, Yaan, Sichuan province, China; 3 Clinical Laboratory, Huzhong Hispital, Guangzhou, Guangdong province, China; 4 Department of Sanitary Inspection, School of Public Health, University of South China, Hengyang, Hunan province, China; Virginia Tech, United States of America

## Abstract

**Background:**

Small non-coding RNAs (sRNAs) facilitate host-microbe interactions. They have a central function in the post-transcriptional regulation during pathogenic lifestyles. Hfq, an RNA-binding protein that many sRNAs act in conjunction with, is required for *Y. pestis* pathogenesis. However, information on how *Yersinia pestis* modulates the expression of sRNAs during infection is largely unknown.

**Methodology and Principal Findings:**

We used RNA-seq technology to identify the sRNA candidates expressed from *Y. pestis* grown *in* *vitro* and in the infected lungs of mice. A total of 104 sRNAs were found, including 26 previously annotated sRNAs, by searching against the Rfam database with 78 novel sRNA candidates. Approximately 89% (93/104) of these sRNAs from *Y. pestis* are shared with its ancestor *Y. pseudotuberculosis*. Ninety-seven percent of these sRNAs (101/104) are shared among more than 80 sequenced genomes of 135 *Y. pestis* strains. These 78 novel sRNAs include 62 intergenic and 16 antisense sRNAs. Fourteen sRNAs were selected for verification by independent Northern blot analysis. Results showed that nine selected sRNA transcripts were Hfq-dependent. Interestingly, three novel sRNAs were identified as new members of the transcription factor CRP regulon. Semi-quantitative analysis revealed that *Y. pestis* from the infected lungs induced the expressions of six sRNAs including RyhB1, RyhB2, CyaR/RyeE, 6S RNA, RybB and sR039 and repressed the expressions of four sRNAs, including CsrB, CsrC, 4.5S RNA and sR027.

**Conclusions and Significance:**

This study is the first attempt to subject RNA from *Y. pestis-*infected samples to direct high-throughput sequencing. Many novel sRNAs were identified and the expression patterns of relevant sRNAs in *Y. pestis* during *in* *vitro* growth and *in* *vivo* infection were revealed. The annotated sRNAs accounted for the most abundant sRNAs either expressed in bacteria grown *in* *vitro* or differentially expressed in the infected lungs. These findings suggested these sRNAs may have important functions in *Y. pestis* physiology or pathogenesis.

## Introduction

*Yersinia pestis* is the causative agent of plague, which is a deadly fulminant disease. *Y. pestis* is primarily transmitted between fleas and mammals and spread to humans by the bite of an infected flea or by contact with afflicted animals [1]. Pneumonic plague is the most severe form of the disease that occurs if the pathogens colonize and multiply in the lungs. *Y. pestis* can be spread from person to person by infectious respiratory droplets; Without timely treatment, pneumonic plague is almost 100% fatal [2]. To survive a range of different stressful environments, *Y. pestis* likely makes appropriate adaptive responses, primarily reflected by the transcriptional changes of specific sets of genes. Transcriptional regulation of protein-coding genes in response to environmental change is extensively analyzed in *Y. pestis* by array hybridization [3,4,5,6].

Small non-coding RNAs (sRNAs) are identified as regulators of gene expression, which function mostly at the post-transcriptional level through sRNA-mRNA interactions [7,8]. They are usually untranslated with length ranges from 50 to 500 nucleotides. Bacterial sRNAs affect a variety of processes including the adaptive responses or pathogenesis [9,10,11]. Hfq, an RNA-binding protein that many sRNAs act in conjunction with, is required for *Y. pestis* pathogenesis [12,13]. In a recently published study from our lab, we used RNomics to find 43 highly abundant sRNAs in *Y. pestis* under multiple growth conditions *in vitro* [14]. RNA-seq studies are used to discover sRNAs in many pathogenic bacteria during growth *in vitro* [15,16,17,18] or within eukaryotes or reservoir hosts [19,20,21]. RNA-seq is used to identify 150 sRNAs in *Yersinia pseudotuberculosis*, the closely related species of *Y. pestis* [22]. Recent studies have also identified dozens of *Y. pestis* sRNAs expressed under normal growth conditions *in vitro* by RNomics or RNA-seq and preliminarily determined different expression patterns under multiple growth conditions [23,24]. However, information on how *Y. pestis* modulates the expression of sRNAs during infection is largely unknown. The present study use RNA-seq technology to more comprehensively discover the novel sRNAs and to monitor sRNA expression in *Y. pestis* infected lungs of mice.

## Results

### Sequencing of four cDNA libraries from *Y. pestis* grown *in vitro* and during infection

**Figure 1:**
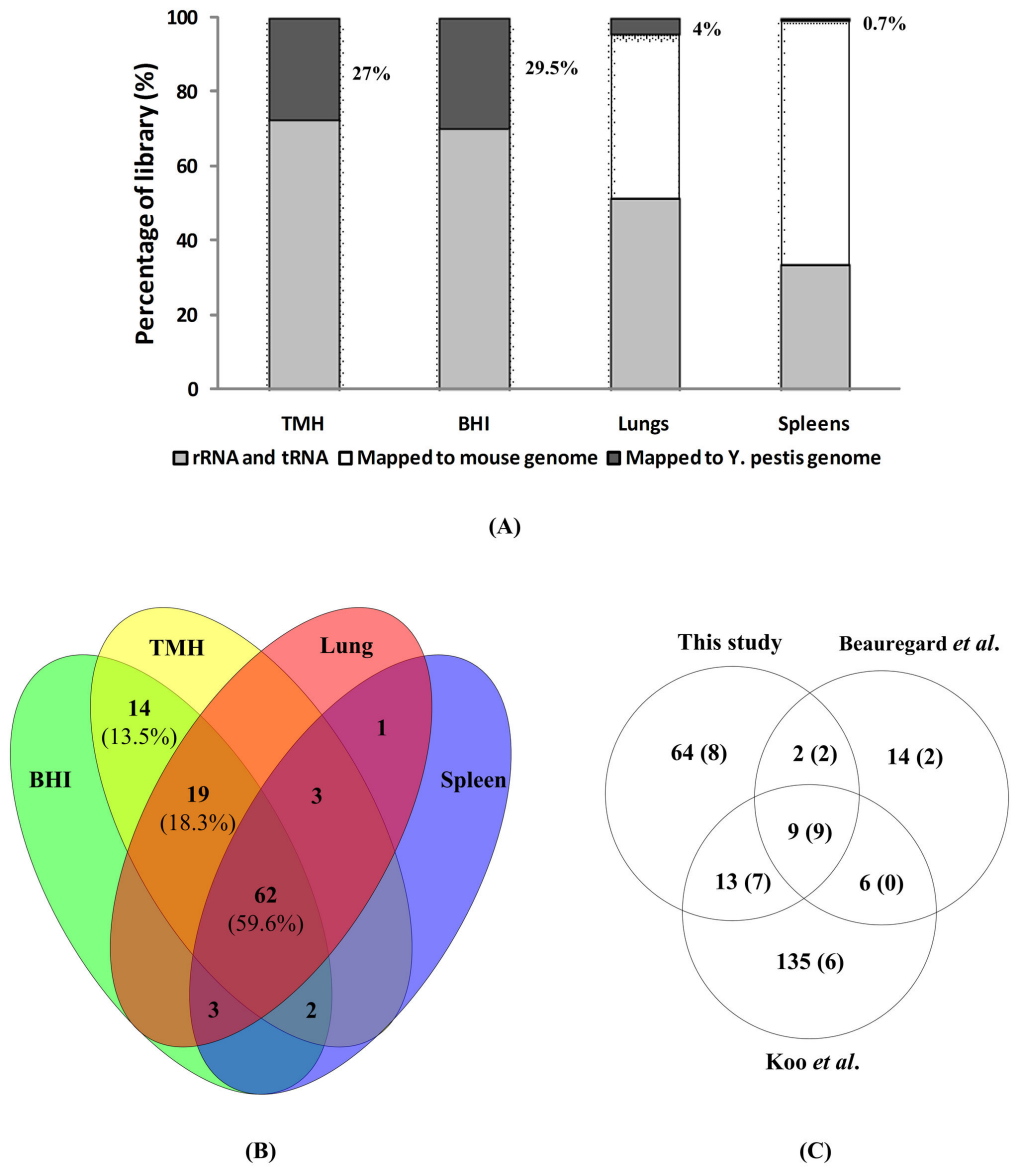
sRNA detection of *Y. pestis* grown *in vitro* or during infection by RNA-seq. (A) Sequence read distribution of the four cDNA libraries. The read percentage of the cDNA libraries from *in* *vitro* (TMH and BHI) and *in* *vivo* (lungs and spleens) samples was indicated, respectively. (B) Sample distribution of sRNAs detected in our study. (C) Comparative analysis of our results with RNA-seq data on *Y. pseudotuberculosis* from Koo et al. [22] and *Y. pestis* from Beauregard et al. [23]. The numbers of sRNAs matching with the Rfam database are in brackets.

To identify sRNAs globally and investigate the sRNA transcriptome proﬁles of *Y. pestis in vivo*, total RNA was separately isolated from bacteria grown in rich, defective nutrition media or infected organs. A chemically defined TMH medium was used as defective nutrition medium and BHI as rich medium. RNAs from bacteria grown in TMH media under five conditions aiming at simulating normal growth or stress processes were pooled together and used as one RNA sample for subsequent experimental procedures as previously described [24]. To enrich our samples, small-fragment RNAs with length of 50 to 500 nt were gel-purified. A mixture of eukaryotic and prokaryotic RNAs was directly subjected to deep sequencing. In total, 67,725,514 cDNA reads allowing less than 1-nt mismatch were mapped to mouse or *Y. pestis* genome. After removing all the mouse-derived sequences, tRNAs, and rRNAs of *Y. pestis*, the remaining 13,260,871 reads with more than one billion bases were aligned to the non-rRNA-tRNA regions of the *Y. pestis* 91001 chromosome (NC_005810) and plasmids (pCD1, NC_005813; pCRY1, NC_005814; pMT1, NC_005815; pPCP1, NC_005816). Among these reads, 8,712,083 and 4,051,853 account for the two samples from bacterial growth *in vitro* and 422,774 and 74,161 for RNA samples from mice lung and spleen, respectively. These fractions represented 27%, 29.5%, 4% and 0.7% of the TMH, BHI, lung and spleen library, respectively (Figure 1A).

### Discovery of the intergenic and antisense RNAs in *Y. pestis*

A sequence file was created for each sample, respectively. The sequence information was displayed in the BedGraph format (http://genome.ucsc.edu/). We extracted intergenic and antisense RNA transcripts with sequential bases forming peaks of high-level expression distinct from those of its flanking regions. A total of 104 sRNAs with predicted promoter or Rho-independent terminator were finally identified (Table S1). Against the Rfam database (http://rfam.janelia.org/), 26 annotated sRNA homologs were detected by our method. The remaining 78 sRNAs appeared to represent a novel set of sRNA candidates in *Y. pestis*. Of the 78 sRNAs, 62 were located within the intergenic region and 16 on the antisense of annotated ORFs. As shown in Figure 1B, 62 sRNAs were detected in four tested samples and 27 sRNAs in three and the remaining 15 sRNAs were present in two samples.

### Comparative analysis with other experimental-based sRNA detections

Given that most studies focus on sRNAs encoded in the intergenic region in bacteria, we compared 88 intergenic sRNAs including 26 known sRNAs and 62 novel sRNAs with two other experimental data [22,23] (Figure 1C). The comparison indicated that only nine previously annotated sRNAs were simultaneously detected by these three studies. Approximately 27% (24/88) of sRNA candidates in this study overlapped with those identified in *Y. pseudotuberculosis* and *Y. pestis* KIM [22,23]. Three fourths (18/24) of these overlapping species were previously annotated sRNAs. Six novel sRNAs detected by two other studies failed to reach the cutoff of this study. Three of these six sRNAs (Ysr11, Ysr73 and Ysr88) were found within ORFs or mostly overlapped with an ORF. These three sRNAs were excluded from further analysis in our study. Three other RNAs (Ysr23, Ysr65 and Ysr145) were not transcribed under our tested conditions. In addition, 9 of 12 intergenic sRNAs identified by cDNA cloning method in our lab [24] could be detected in at least one sample by RNA-seq. The small number of overlapping sRNAs among the three studies may be explained by several reasons. First, sRNA expression patterns are supposed to vary among different species with distinct genetic backgrounds. *Y. pestis* strain 201 (Pgm^+^ pCD1^+^ pMT1^+^ pPst^+^) was used in our study, whereas *Y. pseudotuberculosis* and pCD1 plasmid-cured *Y. pestis* KIM6^+^ were used by Koo et al. and Beauregard et al., respectively. Second, some RNAs are specifically expressed under certain specific conditions that differ among different studies. For example, RyhB1 and RyhB2 known to be induced by iron depletion were detected *in vivo* in our study but missed by two other studies. Third, some sRNAs were failed to be detected in our study, because of the criteria that used in our study, that is, only transcripts with similar ends within at least two samples were kept for further determination of the presence of sRNAs. The criteria are used to identify the stable RNA products under all tested conditions but neglect the occasionally processed products under certain conditions.

**Figure 2:**
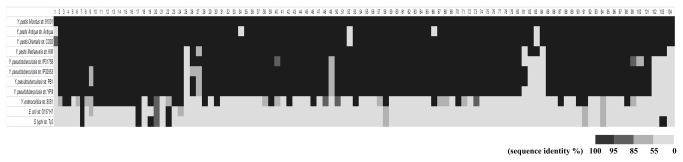
Conservation of 104 sRNAs identified in this study in different *Yersinia* species, *E. coli* and *S* *. typhi* . The heat map shows the conservation of *Y. pestis* sRNAs in 11 genome sequences. Columns and rows represent sRNAs and bacterial genomes, respectively. Sequence identities are indicated with different colors.

To investigate the conservation of the sRNA candidates identified in this study, sequence identity is shown across 11 sequenced genomes including the pathogenic species of *Yersinia* genus (*Y. pestis*, *Y. pseudotuberculosis* and *Yersinia. enterocolitica*) and two enterobacteria (*Escherichia. coli* and *Salmonella* *typhi* ) (Figure 2). Of the 104 sRNAs, only four known sRNAs (RnpB, 6S RNA/SsrS, tmRNA/SsrA and 4.5S RNA) were found to be conserved among all the analyzed genomes. Approximately 30% of these sRNAs (31/104) were found across *Yersinia* species. Approximately 89% (93/104) were conserved in *Y. pseudotuberculosis* and *Y. pestis* genomes and only seven sRNAs (sR001, sR025, sR082, sR083, sR084, sR102 and sR104) were specific to *Y. pestis*. Except for sR001 and sR102 on the chromosome, the five other sRNAs were located on the *Y. pestis*-specific plasmid pMT1 and pPCP1, indicating that acquisition of sRNA may have an important function during the evolution of *Y. pestis* from *Y. pseudotuberculosis*. We also assessed the conservation of the 104 *Y. pestis* sRNAs across the sequenced genomes of 135 *Y. pestis* strains. Approximately 97% of these sRNAs (101/104) share high sequence homology with >95% among more than 80 sequenced genomes of 135 *Y. pestis* strains (Table S2).

### Validation and characterization of selected sRNA candidates

A total of 26 sRNAs were shown to be matched with the Rfam database. Transcription start sites (TSSs) could be predicted by BPROM within 20 nt upstream or downstream of those determined by RNA-seq in more than 70% of 104 sRNAs (74 sRNAs). To test whether the boundaries of novel sRNA transcripts were accurately defined by our methods, Northern blot and primer extension assays were performed to determine the RNA sizes and TSSs of some novel sRNAs, respectively. The expression of seven tested sRNAs was successfully validated and the transcript lengths detected by Northern blot analysis corresponded to the lengths observed by deep sequencing (Figure S1A). We confirmed the presence of two antisense RNAs, namely, sR100 and sR104. The sR100 transcript was transcribed in the opposite orientation covering the ends of two ORFs (*phnI* and *phnJ*) in chromosome, while sR104 is antisense to the YP_PCP04 gene located in plasmid pPCP1 (Figure S1B). The predicted TSS of sR100 transcript agreed well with the 5′ end observed from the deep sequencing. Consistent with this result, the transcript ranged in length between 200 and 300 nt, as detected by Northern blot. Our results showed that sR053 encoded on the same strand of its adjacent gene *ilvB* was less than 200 nt. Given the presence of its own predicted promoter and ρ-independent terminator, sR053 was more likely to be transcribed on its own rather than as the 5′ UTR of *ilvB2*.

**Figure 3:**
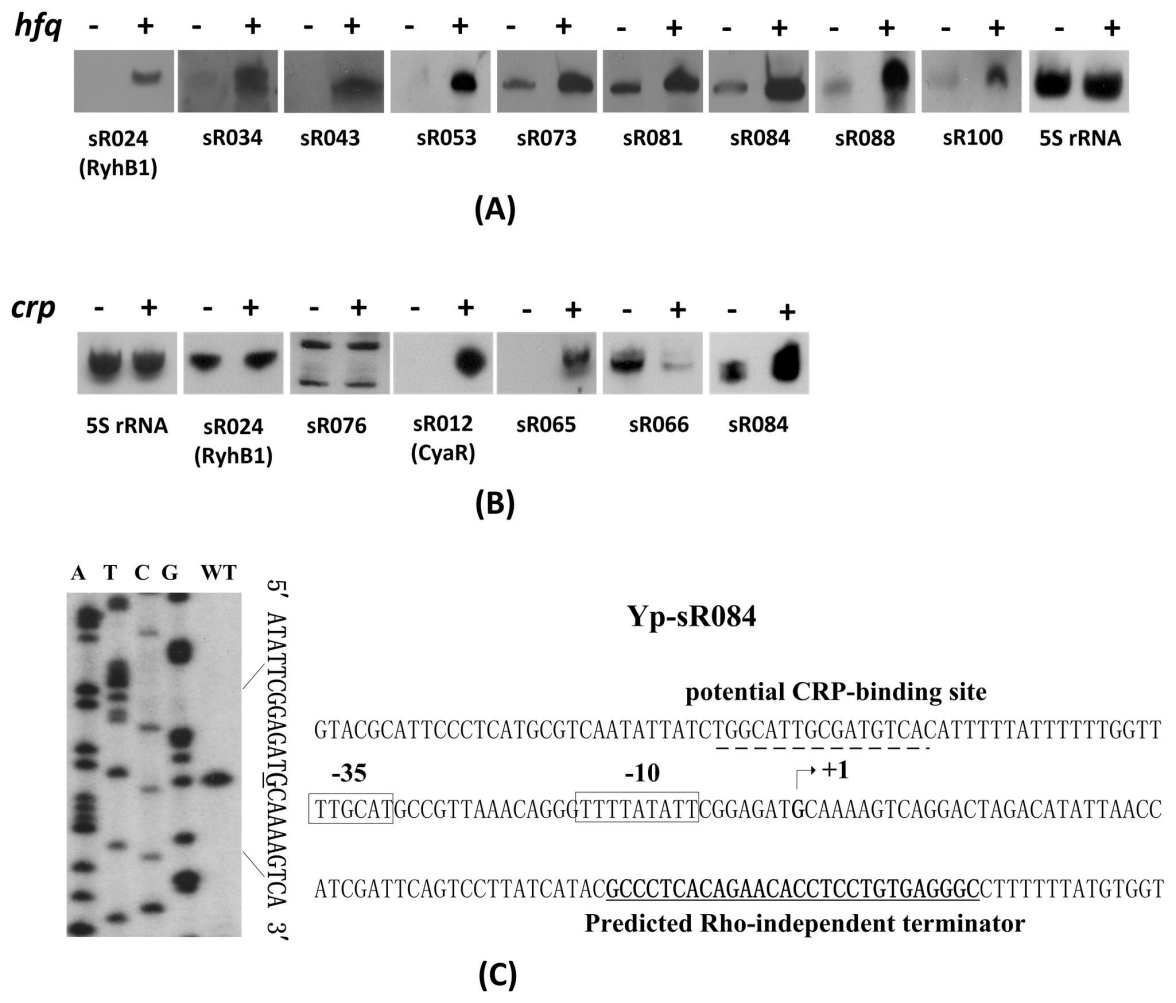
Examination of sRNA expression in *Y. pestis* WT, *hfq* and *crp* mutant strain. (A) Northern blot analysis of sRNA expression in *Y. pestis* WT (hfq^+^) and *hfq* mutant strain (hfq^−^). (B) Examination of sRNA expression in *Y. pestis* WT (crp^+^) and *crp* mutant strain (crp^−^). (C) Promoter analysis of Yp-sR084. TSS determination by primer extension assay is shown on the left panel. Lanes A, T, C and G represent the Sanger sequencing reactions. The transcription start sites were underlined. Organization and sequence features of sR084 are shown on the right panel. The start sites observed by primer extension are indicated by arrows (+1). Putative -10 and -35 regions of the promoters are boxed. The potential CRP binding sites that match CRP consensus [48] are indicated by dashed lines. The predicted *rho*-independent terminator is underlined.

The RNA chaperone Hfq is essential for the regulatory function of *trans*-acting sRNAs at a post-transcriptional level in enteric bacteria [25]. In this study, Northern blot analysis was used to examine the Hfq-dependent or -independent expression of 10 sRNAs. Except for 5S rRNA as a negative control, nine tested RNAs were shown to be Hfq-dependent (Figure 3A). Remarkably, an antisense RNA, sR100, was one of the Hfq-dependent sRNAs. These results supported the original concept that Hfq is required for the stability of a majority of intergenic sRNAs and even antisense sRNAs.

### Three novel sRNAs controlled by the cyclic AMP receptor protein (CRP)

CRP is a global regulator that controls transcriptional initiation of more than 100 promoters in *E. coli* [26]. CRP is also required for the virulence of *Y. pestis*, possibly by regulating certain virulence genes [4]. The 100 bp promoter region upstream of each sRNA detected by RNA-seq was scanned by BPROM. Ten sRNAs containing the conservative CRP-binding sites with a weight score >10 were validated in WT strain and *crp* mutant using Northern blot and seven RNA transcripts were stably detected. Two RNAs, namely, 5S rRNA and RyhB1, were detected in parallel as negative controls because of the absence of predicted CRP-binding sites (Figure 3B). As expected, the previously identified CRP-activated sRNA CyaR/RyeE [27,28] was upregulated by CRP, whereas the negative control RNAs 5S rRNA and RyhB1 were not. Three novel sRNAs (sR065, sR066 and sR084) were regulated by CRP. sR084 species is a highly abundant sRNA encoded in the intergenic region of the pPCP plasmid. The 5′ ends of sR084 determined by primer extension assay were in good agreement with the calculated length from the sequencing data (Figure 3C). A putative binding site for CRP was predicted 56 bp upstream of the TSS. Northern blot showed that this sRNA was obviously downregulated upon the deletion of *crp*, suggesting that this sRNA is positively regulated by CRP. Interestingly, sR066 showed increased expression upon deletion of the *crp* gene in *Y. pestis*, suggesting that it may be negatively controlled by CRP. Further examination showed that the predicted CRP-binding site was overlapping with the potential TSS, which might hinder the transcriptional initiation of this sRNA in the WT strain.

### Confirmation of some differentially expressed sRNA candidates

The expression level of each sRNA was calculated by reads per kb per million reads (RPKM) method. This method eliminates the influence of different sRNA lengths and sequencing discrepancies among different samples on gene expression calculation [29]. Therefore, RPKM values can be used for semi-quantitative comparison of gene expression among samples. To find sRNAs preferentially expressed *in vivo*, the RPKM values of sRNAs with read counts exceeding 500 detected in infected lungs by RNA-seq were calculated. A total of six intergenic sRNAs showed RPKM values at least six times higher in infected lungs than those grown in BHI medium. These sRNAs include five previously identified (RyhB1, RyhB2, CyaR/RyeE, 6S RNA/SsrS and RybB) and a novel sRNA sR039. RyhB1 and RyhB2 were confirmed to be highly expressed *in vivo* by Northern Blot analysis [30]. By contrast, four sRNAs, including CsrB, CsrC, 4.5S RNA and sR027, were downregulated within the infected lungs compared with those *in vitro*. However, CyaR and 4.5S RNA failed to be detected by PCR and Yp-sR039 was inconsistent with the results obtained with RNA-seq. We confirmed the induction or repression of seven sRNAs *in vivo* and *in vitro* using quantitative PCR. In spite of the similar tendency of differential expression ratios shown between qPCR and RNA-seq analysis, more than 10-fold reduction existed in their fold changes detected by qPCR compared with those by RNA-seq method (Table 1).

**Table 1:** Selected differentially expressed sRNAs for RNA-seq and qPCR detection.

**ID**	**Annotated^a^**	**Length (nt**)	**Samples detectable by using RNA-seq**	**Fold changes (Lung/BHI**)**^b^**	
				**RNA-seq**	**qPCR**
sR009	RyhB2	108	BHI, TMH, Lung, Spleen	112.9	15.2
sR012	CyaR/RyeE	93	BHI, TMH, Lung, Spleen	31.9	-
sR017	6S RNA/SsrS	184	BHI, TMH, Lung, Spleen	7.1	2.0
sR023	RybB	86	BHI, TMH, Lung, Spleen	13.5	1.9
sR024	RyhB1	110	Lung, Spleen	>100	8.1
sR039	-	232	BHI, TMH, Lung, Spleen	16.5	0.5
sR003	CsrB	323	BHI, TMH, Lung, Spleen	-7.6	-3.2
sR026	CsrC	386	BHI, TMH, Lung, Spleen	-40.6	-7.2
sR027	-	284	BHI, TMH, Lung, Spleen	-10.7	-2.5
sR020	4.5S RNA	135	BHI, TMH, Lung, Spleen	-10.7	-

a sRNAs that could be annotated by Rfam database were named as is; N represents “unannotated”.

b Fold changes (lung/BHI) represent the abundance in *Y. pestis* within the infected lungs relative to that grown in BHI medium.

**Figure 4:**
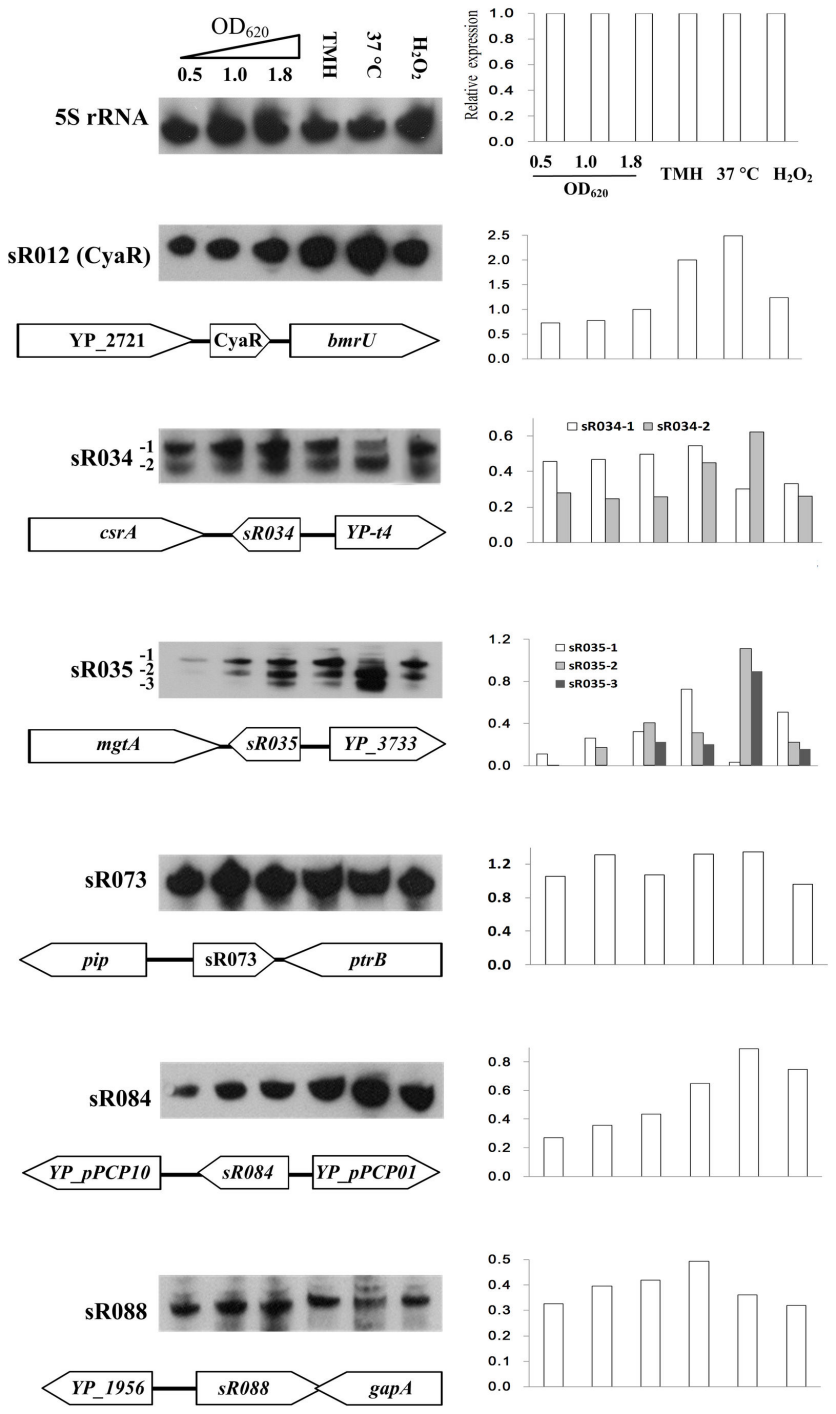
Northern blot analysis of highly abundant sRNAs in *Y. pestis* WT strain grown *in vitro*. The specific RNA probe was used in the Northern Blot analysis to detect each candidate RNA extracted from *Y. pestis* grown at different growth phases (OD_620_ = 0.5, 1.0 or 1.8) or under defective nutrition (TMH), temperature upshift (37 °C) and oxidative stress (H_2_O_2_). The Northern blot results are shown on the left panel. Hybridization signals of Northern blot were quantified by the Quantity One software and normalized to those of 5S rRNA in each biological condition. The relative band intensities are plotted in the bar graphs shown on the right panel. Double or ternary bars for sR034 and sR035 correspond to the expression levels of the two or three RNA species detected for each transcript.

To obtain the expression patterns of differentially regulated sRNAs *in vitro*, Northern blot analysis was used to measure the expression levels of six sRNAs in *Y. pestis* under various conditions, representing distinct growth phases or environmental cues (Figure 4). According to the results of Northern blot analysis, expressions of two sRNAs, namely, sR073 and sR088, were barely affected by the tested conditions. Surprisingly, the rest of the four sRNAs (sR034, sR035, CyaR and sR084) displayed a temperature-dependent expression pattern. In addition, the known sRNA, CyaR/RyeE, was upregulated upon nutrient limitation, whereas the pPCP plasmid-encoded sRNA, sR084, was slightly upregulated upon oxidative stress. Accumulation in post-exponential growth phase was found for the sR035 transcripts. Noticeably, both sR034 and sR035 transcripts possessed more than one band, which were also detected by RNA-seq, thereby excluding the possibility of cross-hybridization. Accumulation in post-exponential growth phase was observed for the sR035 transcripts.

**Figure 5:**
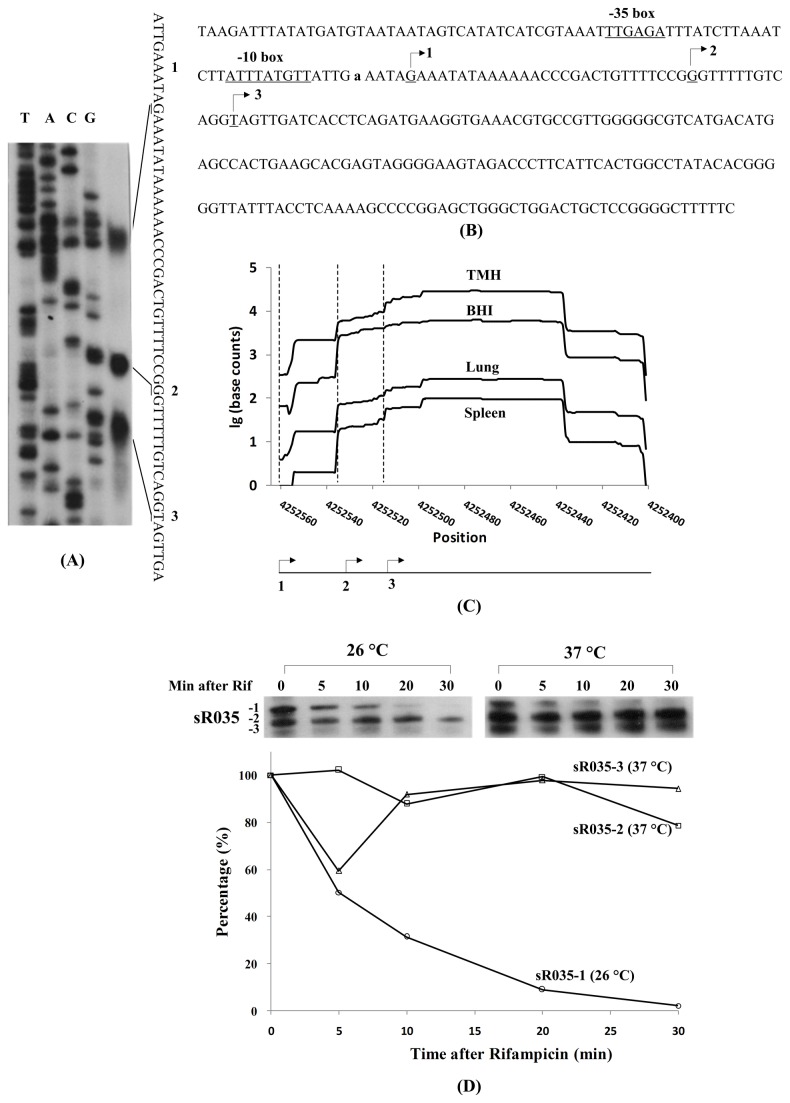
Characterization of sR035 in *Y. pestis* WT 201 strain. Transcription start site determination by primer extension assay. Total RNA mixtures from the culture of *Y. pestis* strain 201 grown in BHI at 26 °C and 37 °C were used to determine TSS by primer extension assays. Lanes T, A, C, and G represent Sanger sequencing reactions. The transcription start sites are underlined. (B) Organization of sequences of sR035. The start sites determined experimentally are indicated by bended arrows 1, 2 and 3. Predicted -10 and -35 regions of the promoters are underlined. The lowercase “a” is the TSS determined by RNA-seq. (C) Distribution of base counts of sR035 among four RNA samples determined by RNA-seq data. The bended arrows 1, 2, 3, and the corresponding vertically broken lines represent the position of TSSs determined by primer extension assays. (D) Measurement of *Y. pestis* sR035 stability at 26 °C and 37 °C. *Y. pestis* strain 201 was grown to middle exponential phase in BHI at 26 °C or 37 °C. Rifampicin was added to the cultures and total RNAs were extracted at the time indicated and analyzed by Northern blot analysis (upper panel). The graph shows the relative amount of sR035 remaining at each time point from Northern blotting results as determined by Quantity One software (lower panel). The percentage of the remaining sR035-1 was calculated based on the Northern blot result from total bacterial RNA of *Y. pestis* grown at 26 °C. The percentages of both sR035-2 and sR035-3 were calculated based on those results at 37 °C.

Northern blot experiments showed that both sR034 and sR035 transcripts were regulated by temperature alteration consistent with the RNA-seq results (Ysr59 and Ysr104) from Lathem’s lab [22]. Interestingly, the shorter species were present in greater amounts in bacteria grown exponentially at 26 °C and then transferred to 37 °C for 3 hr. The 5′ ends of three transcripts were determined by the primer extension assay (Figure 5A and B), which is roughly agreement with that of RNA-seq (Figure 5C). However, due to no predicted promoter elements such as -10 and -35 box, two shorter species of sR035 might be the result of the posttranscriptional processing of the longest transcript at the 5' end. We also measured the RNA stability of these three RNA species expressed at 26 or 37 °C by Northern blot after rifampicin treatment. Quantification of the data showed that the two shorter RNA species were more stable than that of the longest RNA (Figure 5D).

### sRNAs potentially involved in *Y. pestis* pathogenesis

To investigate the potential roles of sRNAs in *Y. pestis* pathogenesis, five annotated sRNAs (RyhB1, RyhB2, CyaR, 6S RNA/SsrS and tmRNA/SsrA) were induced more than four fold *in vivo* compared with *in vitro*. In addition, six novel highly abundant sRNAs (sR034, sR035, sR055, sR073, sR084 and sR088) were chosen to be subjected to subcutaneous and/or intranasal inoculation. The *tmRNA*/*ssrA* deletion mutant showed the obviously decreased virulence in mice compared with that in the WT strain after subcutaneous injection (Figure S2), which is consistent with previous studies [31]. This result suggested that tmRNA/SsrA are essential for *Y. pestis* virulence in mice through different routes. However, no obvious attenuations in mice virulence were found in other sRNA deletion mutants after either subcutaneous or intranasal inoculation (Figure S2 and data not shown).

## Discussion

To gain understanding of the potential functions of non-coding RNAs in *Y. pestis* adaptation to the host environment, we used RNA-seq technology to analyze the expression profiles of *Y. pestis* sRNAs expressed *in vitro* and in the lungs of mice. To ensure that the identified sRNAs were reliable and unbiased, we used RNA-seq data from at least two RNA samples isolated from bacteria grown *in vitro* or *in vivo* to determine the presence of any RNA. A total of 26 sRNAs experimentally verified in *E. coli* and other bacteria were detected with approximately full length in this study. The lengths of several novel transcripts estimated from Northern Blots were roughly in agreement with that determined by RNA-seq. This finding suggested that the criteria used in this study were feasible finding sRNAs with at least high abundance or moderate abundance expressed under two or more conditions.

RNA-seq was proven to be competent in detecting known sRNAs and in discovering novel sRNAs differentially regulated *in vivo*. Approximately 60% of 104 identified sRNAs were independently detected in all four samples. However, 14 sRNAs were only detected *in vitro* but not *in vivo* because of the small amount of bacterial RNA obtained from lungs and spleens. The known sRNAs accounted for the most abundant sRNAs either expressed in bacteria grown *in vitro* or differentially expressed in the infected lungs. This finding suggested these known sRNAs may have play important functions in *Y. pestis* physiology or pathogenesis. Sixteen novel sRNAs expressed at least partially antisense to the known or predicted protein-coding genes. Most antisense transcripts were less abundant than those from the intergenic regions, which may indicate that antisense RNAs can economically function as regulators by perfectly base-pairing with their targets.

Identification of differentially expressed sRNAs derived from *in vivo* or different growth conditions can provide clues for deciphering the mechanism of transcriptional regulation and physiological response. For example, four known sRNAs (RyhB1, RyhB2, CyaR/RyeE and 6S RNA/SsrS) were upregulated *in vivo* and three known sRNAs (CsrB, CsrC and 4.5S RNA) were downregulated. RyhB and CyaR/RyeE are induced upon iron limitation and nutrition deficiency, respectively [32,33]. Therefore, their overexpressions may reflect the status of iron and nutrition limitation in mice lungs. RyhB1 and RyhB2 upregulated in intracellular bacteria influence the capacity of *Salmonella* to restrain intracellular growth [21]. 6S RNA/SsrS, a regulator of RNA polymerase, contributes to intracellular survival and multiplication in other bacteria [34,35]. CsrB and CsrC RNA bind multiple copies of CsrA. CsrA is a protein that post-transcriptionally regulates central carbon flux, biofilm formation and infectious process in some bacteria [36,37,38]. CsrB and CsrC are essential for the initial phase of the infection in *Yersinia* species [39]. 4.5S RNA, an RNA component of the signal recognition particle, is required for the virulence of *Streptococcus* [40]. Moreover, the regulation of sRNAs responded to different growth conditions, suggesting a biological function under these conditions. The most interesting sRNAs detected in this study are sR035 and sR084. TargetRNA prediction showed that sR035 pairs with the SD region and transcription initiation site of *ymoA*, which encodes a thermo-sensitive regulator and sR084 with that of *fur*, ferric uptake regulator, which involves in intracellular iron homeostasis in bacteria. Further efforts should be carried out to determine whether direct interactions occur between RNA and its potential target.

## Materials and Methods

### Bacterial growth conditions

*Y. pestis* strain 201 is avirulent in humans but highly virulent in mice. This bacterium belongs to a newly established biovar, called *Microtus* [41]. The genome contents of strain 201 were identical to *Y. pestis* strain 91001 according to our previous DNA microarray-based comparative genomic analysis [42]. Two growth conditions *in vitro* including nutrient enrichment and deficiency were used in our study. For the nutrient enrichment condition, bacteria were grown at 26 °C in BHI medium with 2.5 mM CaCl_2_ to mid-exponential phase (OD_620_ = 1.0). For growth conditions of nutrient deficiency, bacteria were grown in a chemically defined TMH medium [43] under five growth conditions. These growth conditions were as follows: exponential and stationary phases, iron starvation, calcium deprivation, and low magnesium.

The intranasal mouse model of primary pneumonic plague was developed as previously described [44,45]. *Y. pestis* strain 201 was grown to mid-exponential phase in BHI medium aforementioned. The bacterial cultures were washed and resuspended in sterile PBS. Pathogen-free 8-week-old female BALB/c mice were lightly anesthetized and inoculated by the intranasal route (*i.n.*) with approximately 1×10^6^ cells of *Y. pestis* in PBS. All infected mice were sacrificed after 48 hrs and the lungs and spleens were surgically removed and suspended in RNAlater Solution for subsequent RNA isolation.

### RNA isolation and size fractionation

Total RNA was extracted from *Y. pestis* grown under different growth conditions *in vitro* described above using the TRIzol Reagent according to the manufacturer’s protocol (Invitrogen). *Y. pestis* RNAs from the five different growth conditions in TMH medium were mixed in equimolar ratios. RNAlater solution was removed from the immersed lungs and spleens, followed by homogenization in the TRIzol Reagent. Lung or spleen-derived RNA samples were pooled from 40 infected mice. In total, four RNA samples from TMH, BHI, and infected lungs and spleens were obtained and used for the subsequent procedure. To eliminate DNA contamination, RNA samples were treated twice with DNase I (Fermentas). The total RNA samples were size-fractionated on a 6% TBE-Urea polyacrylamide gel. RNA fragments with length of 50 nt to 150 nt and 130 nt to 500 nt were individually isolated, quantified following gel filtration, and ethanol precipitated.

### cDNA library construction and RNA sequencing

The four fractionated RNA samples described above were subjected to cDNA library construction and deep sequencing performed by LC Sciences LLC, USA. Briefly, RNA fragments with length of 50 nt to 150 nt were dephosphorylated and ligated with 3′ RNA adaptor. The resulting ligated RNA fragments were reversed-transcribed and ligated with 5′ DNA/DNA adaptor. RNA fragments with length of 130 nt to 500 nt were reversed transcribed to single-stranded cDNAs with S-hex primer. The 5' DNA/DNA adaptor was ligated to the 5′ end of the cDNAs, respectively. The adaptor-ligated cDNAs derived from two RNA fragments were amplified by PCR with adaptor-specific primers. A total of four cDNA libraries were constructed and nucleotide sequences were determined with Illumina/Solexa Genome Analyzer system (Illumina, San Diego, CA) that generates 75-nt paired-end reads. The RNA-seq data was deposited in the Sequence Read Archive of NIH (SRA accession number SRS430163, SRS430170, SRS430174 and SRS430175).

### Determination of sRNA candidates and their expression levels

**Figure 6:**
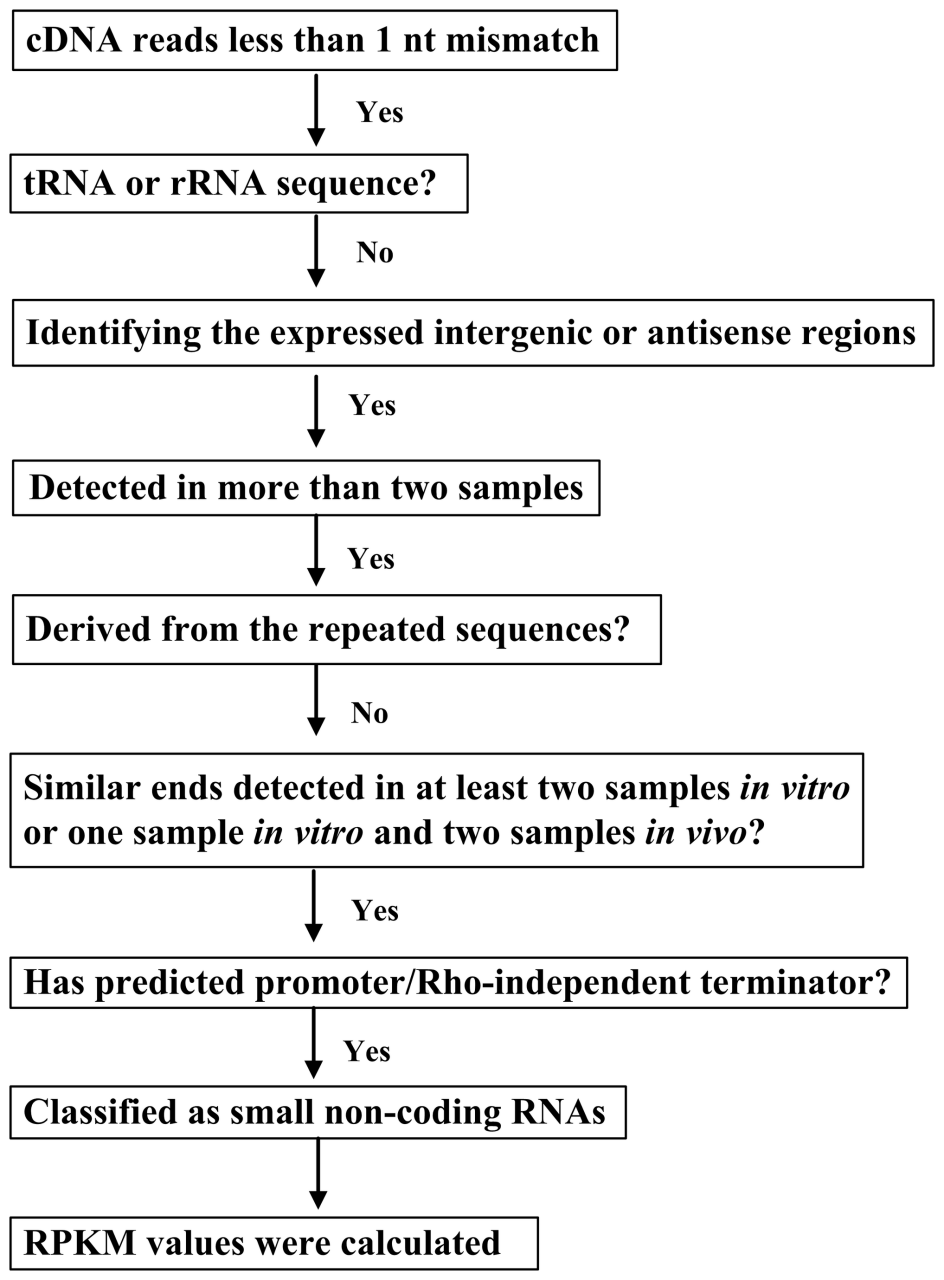
Strategies of sRNA identification in *Y. pestis*.

The steps of RNA-seq data analysis is shown in Figure 6. The abundance of an active transcript is supposed to form a peak region, which is distinguished from its flanking regions. Therefore, the distinct peak regions with expression level above a threshold of at least twofold of the average coverage were separately extracted as distinct RNA transcripts. A total of 7,273 peaks located within the genes were discarded, the remaining 3,806 peaks from the intergenic region or on the antisense strand of genes belong to 1,537 transcripts. Among these transcripts, 774 detected in only a single sample out of the four independent samples were excluded from further analysis. Additionally, 110 repeated sequences including the flanking sequences of rRNA and tRNA genes, insertion sequences and other multi-copy sequences, were further removed. To exclude the possibility of some transcripts as unstable degradation products of RNA, the genomic ends of the remaining transcripts were scanned among all the sequencing data of four samples. Only transcripts with similar ends within at least two samples *in vitro* or one sample *in vitro* and two samples *in vivo* (i.e., the number of differential nucleotides in both ends is within 10% of the maximum transcript in size) were retained for further analysis. However, RyhB1 was maintained because it is highly abundant in two RNA samples *in vivo*. Blastx analysis was performed to identify the novel protein-coding sequences, which were further removed. Bacterial promoter regions were predicted by BPROM (http://linux1.softberry.com/) and Neural Network Promoter Prediction program (http://www.fruitfly.org/seq_tools/promoter.html). Rho-independent transcription terminators were identified using the TransTermHP program [44]. The sequences without predicted promoter and terminator were further removed, thus reducing the number of the sRNA candidates to 104. All the sequences were extracted and searched against the Rfam database, a collection of sequence alignments and profiles of noncoding RNA families [46]. Finally, RPKM was used to estimate the expression levels of all the resulting sRNAs in *Y. pestis* expressed under four different conditions.

### Northern blot analysis

Northern blot analysis was carried out using a DIG Northern Starter Kit (Roche) following the manufacturer’s protocol as described by Beckmann et al. [47]. Total RNA samples (5 µg) were denatured at 70 °C for 5 min, separated on 6% polyacrylamide-7 M urea gel, and transferred onto Hybond N^+^ membranes (GE) by electroblotting. The membranes were UV cross-linked and pre-hybridized for 1 hr, and DIG-labeled RNA probes were added. The membranes were then hybridized overnight at 68 °C in a DIG Easy Hyb according to the manufacturer’s protocols. RNA probes were synthesized by *in vitro* transcription using T7 RNA polymerase. RNA was immunologically detected and scanned according to the instructions.

### Primer extension assay

To determine the TSSs of primary sRNA candidates, primer extension assay was performed using a primer extension system-AMV reverse transcriptase kit (Promega) previously described [48] with slight modifications. Total RNA (10 µg) was reverse-transcribed with ^32^P-labelled primer. cDNA products were subjected to electrophoresis in a 6% polyacrylamide-8 M urea gel. The gel was analyzed by autoradiography (Kodak film). To serve as sequence ladders, sequencing reactions were also performed with the same primers used for primer extension, using an fmol DNA cycle sequencing system (Promega).

### Quantitative RT-PCR (qPCR)

cDNA was synthesized from RNA using the ThermoScrip RT-PCR System (Invitrogen) for gene-specific cDNA synthesis. qPCR was performed in duplicate for each RNA sample using the TransStart^TM^ Green qPCR SuperMix UDG (TransGen Biotech) with an appropriate cDNA dilution as a template. Control reactions were carried out in parallel without a reverse transcriptase. 16S rRNA was used as the internal standard to normalize the expression levels of the tested sRNA candidates.

### Construction of sRNA deletion mutants

*Y. pestis* sRNA deletion mutants were constructed by λ-Red or suicide plasmid homologous combination systems as previously described [12,49]. For the λ-Red method, the sRNA deletion mutants were constructed by replacing the sRNA gene with the *kan* cassette. For the suicide plasmid method, fused PCR products containing 500 nt to 600 nt upstream and downstream of sRNA were cloned into pGMB151 plasmid and transferred electroporately into *Y. pestis*. The markless deletion strain was obtained after incubation on LB plates with streptomycin, followed by those on LB plates with 5% sucrose.

### Mouse infections

*Y. pestis* WT strain 201 and the sRNA mutants were grown in BHI medium at 26 °C overnight and transferred to 37 °C for 1 hr. The bacterial cultures were washed and diluted in 0.85% NaCl. A group of 10 6-week-old BALB/c mice were subcutaneously injected with ~100 cells of bacteria. Mortality was recorded daily for 14 days. All mouse experiments were carried out according to the Guidelines for the Welfare and Ethics of Laboratory Animals of Beijing. The approval number authorized by Committee of the Welfare and Ethics of Laboratory Animals, Academy of Military Medical Sciences, is scxk-(jun)-2007-004. BALB/c mice were lightly anesthetized and infected with *Y. pestis*. All infected mice were sacrificed BALB/c mice were finally anesthetized and sacrificed.

## Supporting Information

Figure S1 **Validation of seven putative novel sRNAs by Northern Blot.** (A) Northern blot results of RNAs isolated from *Y. pestis* strain 201 grown in BHI to exponential phase are shown. The sizes of the different transcripts inferred from RNA-seq are shown at the bottom. (B) The schematic diagrams of the corresponding candidate sRNAs (blue arrows) and their adjacent genes (black arrows) are shown. Gene length is not proportionally indicated.
(TIF)

Figure S2 **Survival of mice subcutaneously infected with 100 CFUs (A) or intranasally infected with 5 × 10^4^ CFUs (B) of *Y. pestis* WT and sRNA deletion strains.**
(TIF)

Table S1 **The information on 104 sRNAs in *Y. pestis* identified by RNA-seq analysis.**
(XLS)

Table S2 **Conservation of 104 sRNAs in the sequenced genomes of 135 *Y. pestis* strains.**
(XLS)
