# Supplementary figures and images for: Determination of sRNA Expressions by RNA-seq in Yersinia pestis Grown In Vitro and during Infection
Source: PLoS One. 2013 Sep 11;8(9):e74495. doi: 10.1371/journal.pone.0074495 (PMC3770706; doi:10.1371/journal.pone.0074495)

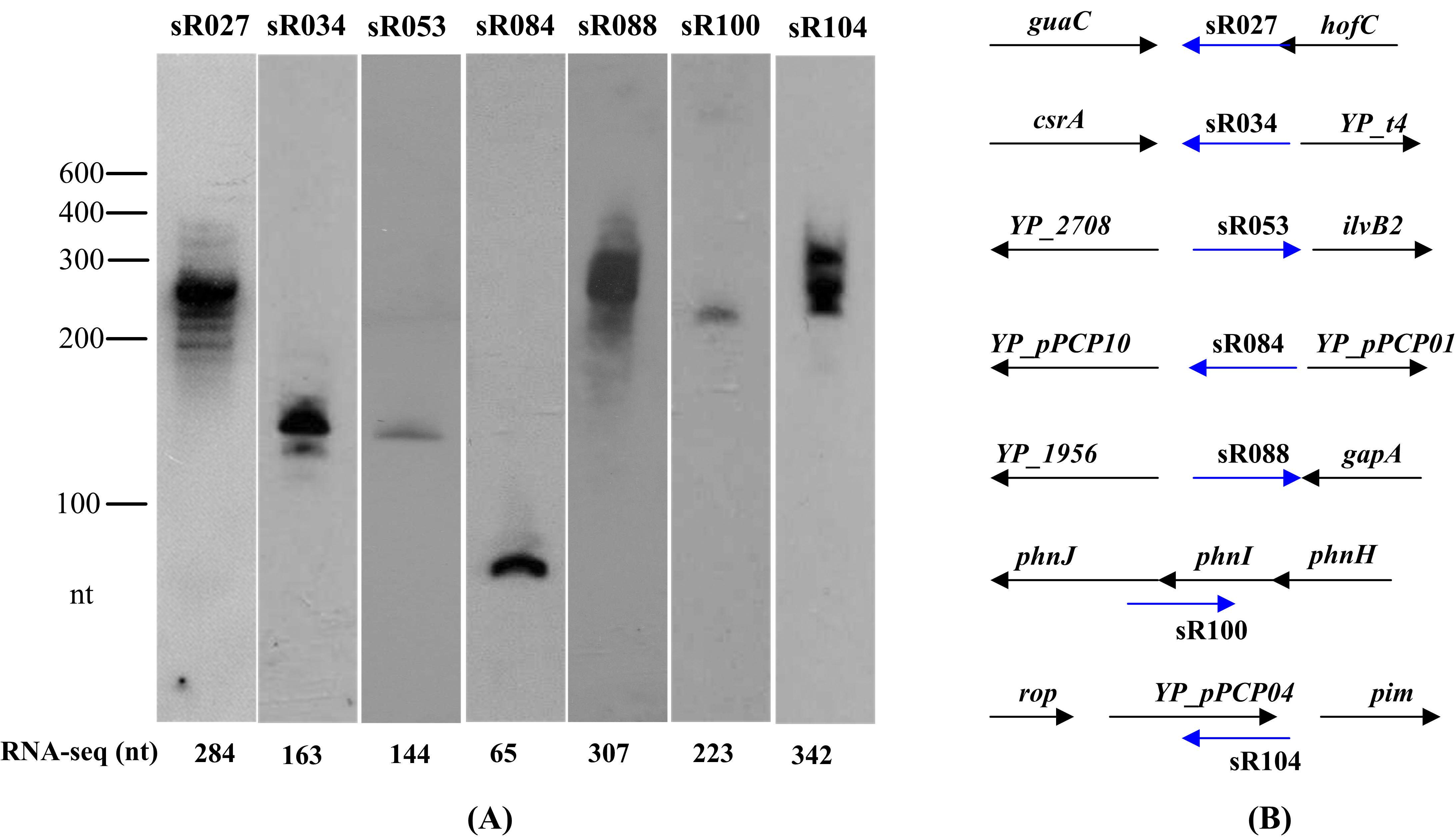

Supplement: Figure S1 — Validation of seven putative novel sRNAs by Northern Blot. (A) Northern blot results of RNAs isolated from Y. pestis strain 201 grown in BHI to exponential phase are shown. The sizes of the different transcripts inferred from RNA-seq are shown at the bottom. (B) The schematic diagrams of the corresponding candidate sRNAs (blue arrows) and their adjacent genes (black arrows) are shown. Gene length is not proportionally indicated. (TIF) [file pone.0074495.s001.tif]

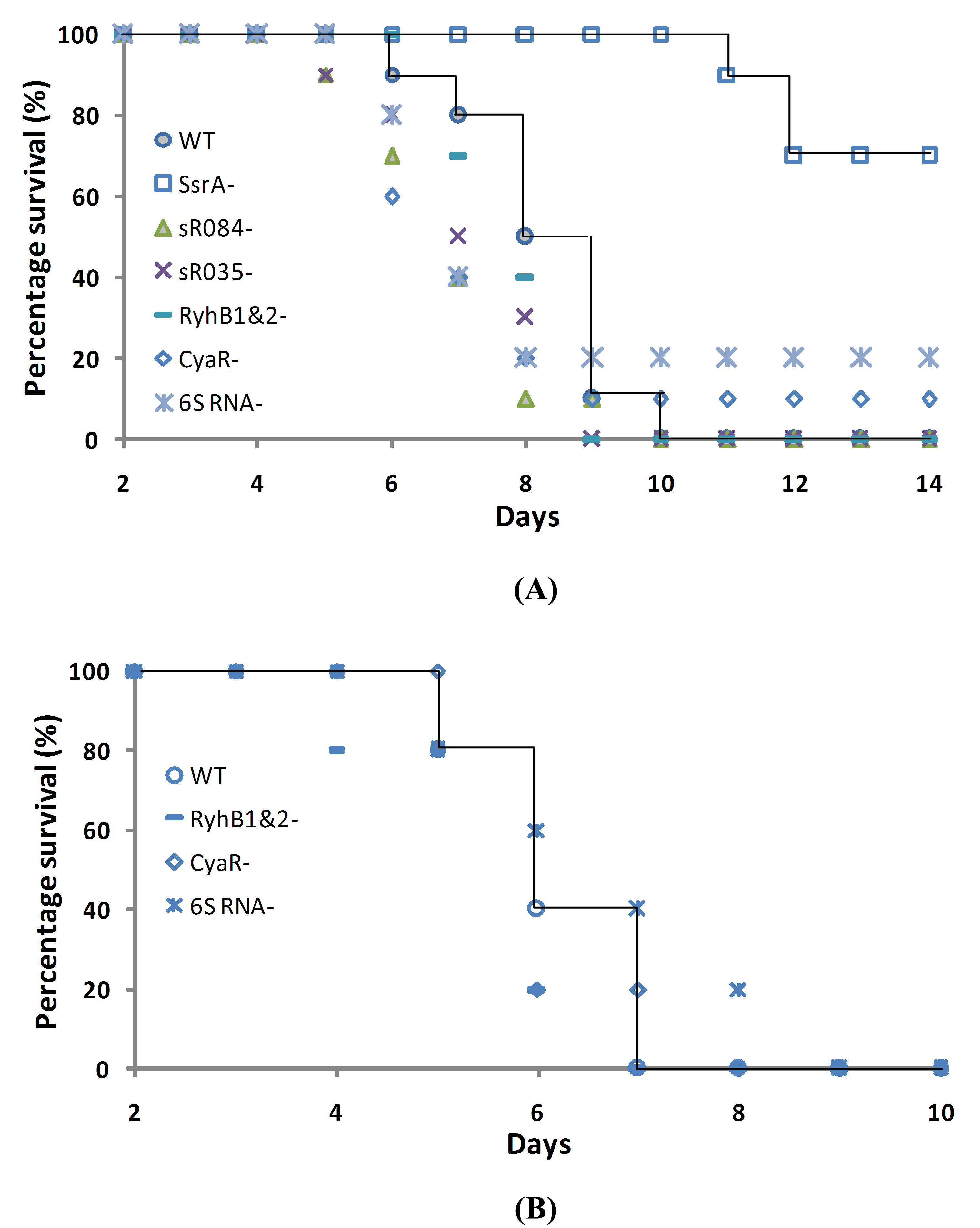

Supplement: Figure S2 — Survival of mice subcutaneously infected with 100 CFUs (A) or intranasally infected with 5 × 10^4^ CFUs (B) of Y. pestis WT and sRNA deletion strains. (TIF) [file pone.0074495.s002.tif]
